# Sleep Deprivation Predisposes Gujarati Indian Adolescents to Obesity

**DOI:** 10.4103/0970-0218.55282

**Published:** 2009-07

**Authors:** Wasim A Shaikh, Minal Patel, SK Singh

**Affiliations:** Department of Physiology, Pramukhswami Medical College, Karamsad-388 325, Gujarat, India

**Keywords:** Gujarati Indian adolescents, obesity, sleep duration

## Abstract

**Background and Aim:**

Recent studies on various populations indicate that lack of sleep is one of the potential risk factors predisposing the youth to obesity. Since there is a significant rise in obesity among Indian youth and because research indicating the role of sleep in development of obesity among Indian population is scant, the current study was undertaken to assess the effect of sleep duration on adiposity among Gujarati Indian adolescents.

**Materials and Methods:**

A randomized cross-sectional study was done on 489 voluntarily participating Indian adolescents in the age group of 16-19 years. Participants were grouped into two categories 1). Adequate Sleep Duration at Night (more than seven hours, ASDN) and 2) Inadequate Sleep Duration at Night (less than seven hours, IASDN) as reported by the participants. The participants were later assessed for adiposity in terms of BMI, BF %, FM, FMI and waist circumference, meal frequency per day and physical activity status.

**Results:**

In both boys as well as girls, the BMI, BF%, FM and FMI were significantly lower in the ASDN group than the IASDN group. However, there was an insignificant difference in the meal frequency and physical activity status between the ASDN and IASDN group.

**Conclusion:**

Inadequate sleep duration increases adiposity among Gujarati Indian adolescents but further studies are required to find out the mechanisms through which sleep affects adiposity in this population.

## Introduction

A phenomenal rise has been observed in the prevalence of overweight and obesity among Indian youth.(1) This has been attributed to nutritional transition in India, characterized by a shift in the diet content towards a high fat and high sugar diet.(2) However, recent studies conducted in various parts of the world indicate that sleep has evolved as a significant determinant of body composition besides conventional factors like physical activity status, dietary habit and genetic inheritance that determine the body composition.(3–5) These studies indicate that lack of adequate sleep predisposes the children to overweight and obesity. Sleep has been known to influence the physical and emotional well being of adolescents by causing substantial biological and psychosocial changes.(6)

The current study was undertaken to assess the effect of sleep duration on adiposity among the Gujarati Indian adolescents since ethnic differences occur in the aetiopathogenesis of obesity as indicated by various studies,(7) but there are scant reports available on Indian population indicating the influence of sleep on adiposity among adolescents. The study also intended to find the effect of sleep duration on the feeding habit and physical activity status so as to delineate the probable mechanism involved through which sleep duration may influence body composition.

## Materials and Methods

A randomized cross-sectional study was conducted following approval from the human research ethical committee of the institute and obtaining informed consent from the participants or their parent/guardian. Multi-stage sampling was used for recruitment of participants into the study. The study was conducted on the students of age group 16-19 years, studying at schools and colleges in the local population, irrespective of socio-economic class and who had attained the Tanner Stage 4 by self-reporting.(7) A total of 489 subjects (Boys = 297, Girls = 192) were studied after measuring the sample size on the basis of a pilot study using PEPI software (Version 4.0, J H Abramson, P M Gahlinger, 1993-2001).

## Sleep duration at night

The participants were asked to self-report the number of hours for which they slept during most of the nights, in a week, for the last one-year. The subjects reported the sleeping hours from the time of going to bed to the time they woke up in the morning.(3)

## Feeding habit and physical activity status

The participants were asked to report the number of times they eat in a day (inclusive of breakfast, lunch, dinner, snacks) over the last one-year.(8) Participants also reported their physical activity status for the last one year using the NASA/Johnson Space Center Physical Activity Rating scale.(9)

## Body composition

The body composition was assessed in a standardized state of clothing. The body weight (Wt) was recorded bare footed to the nearest 0.5 kg. The height was measured using meter scale without footwear to the nearest 5 cm. BMI was calculated as the weight (kg) divided by the square of height (m^2^).(10) Body Fat Percentage (BF %) and Total Body Fat Mass (FM) were assessed by bioelectrical impedance technique using Omron Body Fat Monitor HBF -302.(11) Handles of the device contain plate electrodes, which are held by both hands with arms outstretched and parallel to the ground while standing erect. The Omron Body Fat Monitor sends a weak electrical current of 0.5Mv through the body from one electrode and receives the resistance encountered by the current as the current flows through the body. It then calculates the BF% and FM of the individual using the impedance encountered in consideration with the age, gender, height and weight of the person. Fat Mass Index (FMI) was calculated as the Fat Mass (kg) divided by the square of height (m^2^). Waist circumference was measured at the mid point between the lower costal margin and the highest point on the iliac crest to the nearest 0.5 cm at the end of normal expiration.(10)

## Statistical analysis

The subjects were categorized into two groups based on the sleep duration at night: Adequate Sleep Duration at Night (more than seven hours), ASDN and Inadequate Sleep Duration at Night (less than seven hours), IASDN.(3)

The study variables (Body composition, Meal Frequency and Physical Activity Status) of the two groups were then compared using unpaired student's t-test at 95% confidence limit and five per cent confidence interval.

## Results

Tables 1 and 2 show that in both genders, adolescents in ASDN group had a significantly (*P*< 0.05) lower weight, BMI, BF%, FM and FMI as compared to the adolescents in the IASDN group. Waist circumference was also found to be significantly lower in the boys of ASDN group as compared to boys of IASDN group. However, there was an insignificant difference in the meal frequency and physical activity status between the ASDN.

**Table 1:** Comparison of body composition, meal frequency and physical activity status between ASDN and IASDN group of boys

Variables	ASDN N=210	IASDN N=87
Sleep duration at night (Hrs)	7.9 ± 1.1	5.7 ± 0.4*
Meal frequency	3.6 ± 1	3.6 ± 0.93
PA-R	3 ± 1.2	3.2 ± 1.5
Weight (kg)	49 ± 10.5	56 ± 12.3**
Height (m)	1.64 ± 0.08	1.67 ± 0.07*
BMI (kg/m^2^)	18.2 ± 2.8	19.8 ± 3.8**
BF%	16 ± 5.2	17.6 ± 5.8*
FM (kg)	8.3 ± 4.1	10.3 ± 5.4**
FMI (kg/m^2^)	3 ± 1.4	3.6 ± 1.9**
Waist circumference (cm)	65.3 ± 6.8	68.8 ± 9.4**

Values are Mean ± SD;

* *P* < 0.05

** *P* < 0.01; ASDN: Adequate sleep duration at night; IASDN: Inadequate sleep duration at night

**Table 2:** Comparison of body composition, meal frequency and physical activity status between ASDN and IASDN group of girls

Variables	ASDN N=139	IASDN N=53
Sleep duration at night (Hrs)	7.6 ± 0.8	5.8 ± 0.3*
Meal frequency	3.5 ± 0.92	3.7± 1.1
PA-R	1.96 ± 0.98	1.98 ± 1.23
Weight (kg)	45.7 ± 9.4	49.6 ± 9.7*
Height (m)	1.54 ± 0.06	1.55 ± 0.06
BMI (kg/m^2^)	19 ± 3.5	20.4 ± 3.5*
BF%	23.3 ± 7	26.2 ± 5.7*
FM (kg)	11.2 ± 5.6	13.4 ± 5.4*
FMI (kg/m^2^)	4.6 ± 2.2	5.5 ± 2.1*
Waist circumference (cm)	63.5 ± 7	64.9 ± 7.2

Values are Mean ± SD;

* *P* < 0.05; ASDN: Adequate sleep duration at night; IASDN: Inadequate sleep duration at night

## Discussion

The results of this study reveal that sleep duration of less than seven hours is a risk factor for obesity in Gujarati Indian adolescents. Similar findings were reported in the NHANES I survey, where adolescents with self-reported sleep durations at baseline less than seven hours had higher average body mass indexes and were more likely to be obese than subjects with sleep duration of seven hours.(3) A cross-sectional study done on South Indian children in the age group 6-16 years showed that children who slept less than eight and half hours/day had significantly higher odds of being overweight when compared to children who slept more than nine and half hours/day, after adjustments for age, gender, location of stay and socio-economic status.(8)

Several studies are being done to elucidate how sleep deprivation affects adiposity. A recent study has indicated that at least three pathways may be involved which link sleep deprivation with adiposity i.e., alterations in glucose metabolism, up-regulation of appetite and decreased energy expenditure.(12) Studies have shown that the increased adiposity observed in sleep-deprived individuals is due to changes in the plasma concentrations of Leptin and Ghrelin, the hormones that regulate the eating behavior of an individual and a compromise in the Insulin sensitivity.(13,14) A crossover clinical study indicated that sleep restriction was associated with an average reduction in the anorexigenic hormone Leptin, elevations in the orexigenix factor ghrelin and an increase in hunger especially for calorie dense foods with high carbohydrate content.(13)

The current study, however, shows that in the Gujarati adolescents meal frequency seems to be unaffected by the duration of sleep, but it may be possible that the sleep duration may be affecting the eating behavior in other aspects such as the type of food or quantity per meal which have not been studied in the present study.

Though literature indicates that deprivation impairs physical functioning and emotional well being with reduction in motivation and early fatigue,(15) the current study shows that an insignificant difference exists in the physical activity pattern between adolescents having adequate sleep duration and adolescents having inadequate sleep duration.

## Conclusion

This study shows that sleep deprivation (night sleep duration less than seven hours) significantly affects the body composition of the Gujarati adolescents of age group 16-19 years and predisposes them to the risk of overweight and obesity. However, further studies are required to understand the mechanism of increase in fat mass in this population because of sleep deprivation.

## References

[1] Kapil U, Singh P, Pathak P, Dwivedi SN, Bhasin S (2002). Prevalence of Obesity amongst affluent adolescent school children in Delhi. Indian Pediatr.

[2] Shetty PS (2002). Nutritional Transition in India. Public Health Nutr.

[3] Gangwisch JE, Melaspina D, Bodex-Albala B, Heymsfield SB (2005). Inadequate Sleep as a risk factor for Obesity: Analysis of the NHANES I. Sleep.

[4] Yu Y, Lu BS, Wang B, Wang H, Yang J, Li Z Short Sleep Duration And Adiposity In Chinese Adolescents. Sleep.

[5] Snell EK, Adam EK, Duncan GJ (2007). Sleep and the Body Mass Index and Overweight Status of Children and Adolescents. Child Development.

[6] Dahl RE, Lewin DS (2002). Pathway to Adolescent Health: Sleep Regulation and Behaviour. J Adolesc Health.

[7] Novotny R, Going S, Teegarden D, Van Loan M (2007). Hispanic and Asian Pubertal Girls have Higher Android/Gynoid Fat Ratio than Whites. Obesity.

[8] Kuriyan R, Bhat S, Thomas T, Vaz M, Kurpad AV (2007). Television Viewing and Sleep are associated with Overweight among urban and semi-urban South Indian children. Nutr J.

[9] Pate RR, Pratt M, Blair SN, Haskell WL, Macera AA, Bouchard C (1995). Physical activity and public health: A recommendation from the Centers for Disease Control and Prevention and the American College of Sports Medicine. JAMA.

[10] Janssen I, Katzmarzyk PT, Ross R (2002). Body mass index, waist circumference, and health risk: evidence in support of current National Institutes of Health guidelines. Arch Intern Med.

[11] Wong CH, Wong SF, Pang WS, Azizah MY, Dass MJ (2003). Habitual Walking and Its Correlation to Better Physical Function: Implications for Prevention of Physical Disability in Older Persons. J Gerontol A Biol Sci Med Sci.

[12] Knutson KL, Spiegel K, Penev P, Van Cauter E (2007). The metabolic consequences of sleep deprivation. Sleep Med Rev.

[13] Spiegel K, Tasali E, Penev P, Van Cauter E (2004). Brief communication: Sleep curtailment in healthy young men is associated with decreased leptin levels, elevated ghrelin levels, and increased hunger and appetite. Ann Intern Med.

[14] Spiegel K, Knutson K, Leproult R, Tasali E, Van Cauter E (2005). Sleep loss: a novel risk factor for insulin resistance and Type 2 diabetes. J Appl Physiol.

[15] Chen MY, Wang EK, Jeng YJ (2006). Adequate sleep among adolescents is positively associated with health status and health-related behaviors. BMC Public Health.

